# Plasma proteomic analysis of active and torpid greater mouse-eared bats (*Myotis myotis*)

**DOI:** 10.1038/srep16604

**Published:** 2015-11-20

**Authors:** Alexander M. Hecht, Beate C. Braun, Eberhard Krause, Christian C. Voigt, Alex D. Greenwood, Gábor Á. Czirják

**Affiliations:** 1 Leibniz Institute for Zoo and Wildlife Research, Alfred-Kowalke-Straße 17, 10315 Berlin, Germany; 2 Leibniz Institute for Molecular Pharmacology, Robert-Rössle-Straße 10, 13125 Berlin, Germany; 3 Department of Animal Behaviour, Freie Universität Berlin, Takustraße 3, 14195 Berlin, Germany; 4 Department of Veterinary Medicine, Freie Universität Berlin, Oertzenweg 19b, 14163 Berlin, Germany

## Abstract

Hibernation is a physiological adaptation to overcome extreme environmental conditions. It is characterized by prolonged periods of torpor interrupted by temporary arousals during winter. During torpor, body functions are suppressed and restored rapidly to almost pre-hibernation levels during arousal. Although molecular studies have been performed on hibernating rodents and bears, it is unclear how generalizable the results are among hibernating species with different physiology such as bats. As targeted blood proteomic analysis are lacking in small hibernators, we investigated the general plasma proteomic profile of European *Myotis myotis* and hibernation associated changes between torpid and active individuals by two-dimensional gel electrophoresis. Results revealed an alternation of proteins involved in transport, fuel switching, innate immunity and blood coagulation between the two physiological states. The results suggest that metabolic changes during hibernation are associated with plasma proteomic changes. Further characterization of the proteomic plasma profile identified transport proteins, coagulation proteins and complement factors and detected a high abundance of alpha-fetoprotein. We were able to establish for the first time a basic myotid bat plasma proteomic profile and further demonstrated a modulated protein expression during torpor in *Myotis myotis*, indicating both novel physiological pathways in bats in general, and during hibernation in particular.

In order to overcome food limitation and high metabolic energy demands during winter periods, animals in temperate climate zones have evolved strategies such as diet switching, annual migration, winter resting or hibernation. Hibernating species undergo a circannual rhythm between homeothermy (activity) and heterothermy (hibernation), in which the heterothermic hibernation cycle alters between extended phases of deep torpor interspersed by short rewarming phases called arousals^1^. During the torpid phase, which lasts between 6 and 40 days, the metabolic rate drops to 2% of normal coinciding with lowered body temperatures between 10 °C and −2 °C, decreased heart rate and longer breath intervals^1^,^2^,^3^. During arousal, torpor-associated physiological changes are restored to euthermic values for 10–15 hours^1^. As a consequence of metabolic suppression, the immune system is also functionally suppressed during torpor and restored during arousals in order to clear infections^4^.

To describe the mechanism underlying the circannual rhythm of hibernation a two-transition model has been proposed, which designates a transition from homeothermy to heterothermy and another cyclic transition within the heterothermic state between torpor and arousal^5^,^6^. Although the precise genetic and molecular regulation underlying this mechanism remains unclear, it was shown that the switch from homeothermy to heterothermy is associated with a broad differential expression of existing genes rather than with the evolution and expression of hibernation-specific genes^7^,^8^,^9^. These expression differences occur at the protein level in heart^10^, intestine^11^, liver^12^ and skeletal muscles^13^ of ground squirrels (*Ictidomys tridecemlineatus*). In these tissues, increased expression levels of proteins involved in glycolysis, glycogenesis and amino acid catabolism were observed during the active state, while hibernation was characterized by increased expression of proteins involved in fatty acid catabolism. These findings were consistent with the shift from carbohydrates to lipid oxidation during hibernation^14^. Another study investigating the kidney proteome in the same species found evidence for a turnover of plasma proteins alpha-2-macroglobulin, albumin and apolipoprotein during torpor-arousal cycles^15^.

Plasma proteins and their composition in the blood are known to be an important indicator of physiological changes including disease recognition or pathogen infection in humans^16^. Thus hibernation-associated changes in blood plasma protein composition may also be detectable. In Asian chipmunks (*Tamias sibiricus asiaticus*), for example, specific proteins associated with hibernation have been identified in plasma^17^,^18^, while in ground squirrels (*Ictidomys tridecemlineatus*), altered plasma metabolites (e.g. amino acids, regulatory lipids) during the heterothermic hibernation cycle were characterized using non-targeted metabolomic approaches^19^,^20^. A targeted proteomic analysis of the blood serum in hibernating American black bears (*Ursus americanus*) revealed differential expression of proteins involved in immunity, coagulation and bone metabolism^21^. These results provide molecular support for the peculiarities of ursid hibernation, including wound healing and active immune function during hibernation^22^. In contrast to bears, rodent hibernation is associated with immunosuppression^4^, highlighting the importance of research on blood proteomics in small mammalian hibernators given the lack of a universal mammalian hibernation proteomic profile.

Research on mammalian hibernation has focused on rodents. Although there is information available on general regulatory processes from tissue proteomic studies in rodents, targeted analysis of plasma and serum proteome profiles is lacking. Even if the hibernating phenotype between species is similar across taxa, differences in the mechanisms leading to hibernation associated changes are unclear, especially if physiological adaptations during homeothermy are considered. Accordingly, similarities in physiological aspects of hibernation may be expected for rodents and bats, e.g. fuel change from carbohydrate to lipid metabolism, reduction in protein synthesis, among others. However other processes may be unique due to basic differences in their physiology. Bats are able to enter a heterothermic state throughout the year by entering bouts of prolonged torpor also including the summer period^23^ while most hibernating rodents exhibit heterothermy only during the winter season. Bats are also the only mammalian group capable of powered flight, which has selected for increased metabolic capacity and elevated antioxidant levels^24^. Moreover, they are unusually long-lived species^25^, traits which are different from the hibernating rodents studied to date. Furthermore, the reproduction patterns of hibernating bats^26^,^27^ or the role of wing membrane in maintaining the water balance^28^, makes chiropteran hibernation related physiology unique. It has been suggested that some bat species are not immunosuppressed during hibernation as observed in rodents and that bats maintain specific defenses against psychrophilic pathogens such as *Pseudogymnoascus destructans*^29^. Therefore, we hypothesized that some of the regulatory mechanisms of hibernating bats should be distinct from those of rodents.

In order to improve our knowledge on chiropteran hibernation and in small mammalian hibernation in general, we compared the blood plasma proteomic profile of a hibernating European bat species, the greater mouse-eared bat (*Myotis myotis*) by using a two-dimensional gel electrophoresis approach to characterize differential expressed proteins between homeothermic and heterothermic, torpid individuals. In comparison to rodent hibernation we predict for bats similar pattern of protein expression in general regulatory mechanisms but differences in the regulation of proteins involved in specific physiological processes such as immune function or reproduction.

## Materials and Methods

### Ethics statement

All experimental procedures described in the materials and methods section were approved by the animal welfare and ethics committee of the Leibniz Institute for Zoo and Wildlife Research (permit #2010-05-01). All experiments were carried out in accordance with the approved guidelines of the Leibniz Institute for Zoo and Wildlife Research.

### Sample collection

Greater mouse-eared bats (*M. myotis*) were captured in Northern Bavaria (Germany), under the license of regional governments (permits 54-2532.2-9/10, 55.1-8642G062/10 and 55.1-8642-01-17/10). Blood samples from 14 male individuals in the homeothermic active state (n = 7) were captured in September 2010 and torpid individuals (n = 7) during the hibernation period in March 2011. Active individuals were captured using mistnets (Ecotone, Poland), while torpid individuals were picked by hand from the walls of the hibernacula. Blood samples were collected in active bats from the uropatagial vein using sterile needles and by transferring blood droplets into heparinized microcapillary tubes and in torpid bats from the jugular vein using a sterile heparinized needle and syringe. All bats were released at the site of capture after bleeding was completed. Structural and functional immunological measurements were performed on fresh blood while in case of surplus blood the plasma was separated by centrifugation and stored at −80 °C until further analysis.

### 2-Dimensional Fluorescence Difference Gel Electrophoresis (2-D DIGE)

Proteomic profiles were determined from blood plasma samples of 14 *M. myotis* male individuals (7 homeothermic, active individuals and 7 heterothermic, torpid individuals) using 2-D DIGE.

Serum albumin is the most abundant plasma protein in humans^16^ and can impede the detection and quantification of low abundance plasma proteins^30^. Therefore plasma investigation studies often deplete albumin prior to analysis. However the depletion of albumin also can remove untargeted proteins^31^. Albumin depletion was not performed in this study but serum albumin was excluded during mass spectrometry identification.

Total plasma protein concentration was determined using a NanoDrop® and diluted to the required concentration of 0.55 μg protein/μL in labeling buffer [50 mM tris, 5 mM EDTA, 5% v/v glycerol, pH 7.2; final volume = 9 μL] for fluorescent protein labeling using S-Dye300 of the Saturn-2D^TM^ labeling kit (NH DyeAGNOSTICS GmbH, Germany) according to manufacturer´s protocol. An internal standard (IS) consisting of all samples used in each experimental procedure was diluted to the required concentration of 0.55 μg protein/μL in labeling buffer (final volume = 9 μL) and fluorescent labeled using S-Dye200 of the Saturn-2D^TM^ labeling kit.

Labeled samples (per gel: 9µL of an individual S-Dye300-labeled sample + 9µL of S-Dye200-labeled IS) were diluted in 432 µL rehydration buffer [8 M Urea, 1% w/v 3-[(3-Cholamidopropyl)dimethylammonio]-1-propanesulfonate hydrate (CHAPS), 13 mM Dithiothreitol (DTT), 0.5% v/v Servalyt (SERVA Electrophoresis GmbH, Germany)] and loaded on IPG *Blue*Strips pH 3–10 NL/24 cm (SERVA Electrophoresis GmbH, Germany) for active (50 V, 15 h) sample-in-gel rehydration using PROTEAN® IEF Cell tray (Bio-Rad, USA). Isoelectric focusing was performed under following conditions: step 1, 300 V, 150 V/h rapid; step 2, 600 V, 300 V/h rapid; step 3, 1500 V, 750 V/h rapid; step 4, 3000 V, 48000 V/h rapid; step 5, 6000 V, 10000 V/h rapid; step 6, 300 V, 5 h; total 60700 V/h.

Prior to second dimension separation, IPG stripes were equilibrated in equilibration buffer [EB: 6 M Urea, 2% SDS, 0.375 M Tris, 20% v/v glycerol] with first 20 mg/mL DTT for 15 min, followed by EB with 25 mg/mL iodoacetamide (IAA) for 15 min. After equilibration, stripes were placed on 15% SDS gels in 27.5 × 22 cm low fluorescence glass cassettes (NH DyeAGNOSTICS GmbH, Germany) and overlaid with 1% agarose including bromphenol blue. Gel electrophoresis was performed in a SE900 electrophoresis unit (Hoefer Inc., USA) for a minimum of 1900 V/hours and a maximum of 2200 V/hours at 80 mA/ gel, 100 W and 100 V. Imaging of the gels was performed by fluorescence scanning on a Typhoon 9400 Imager (GE Healthcare, USA) at excitation/emission wavelengths of 532/576 nm (S-Dye200) and 633/664 nm (S-Dye300).

To evaluate the expression pattern of protein spots separated by 2-D DIGE, all sample gels were analyzed using the Delta2D software (DECODON, Germany). An IS S-Dye200 image was designated as the master gel based on the largest number of detectable spots, and then connected to all images by a “sample in gel” warping strategy in the Delta2D software. Warping of gels was done by defining matched vectors between distinct protein spots chosen automatically and manually. For expression analysis of protein spots, a fused image of all sample images (S-Dye300; gel images of each sample are shown in supplementary Figure S1) was generated and a consensus spot pattern for normalization against IS images was applied. Matched protein spots present in all sample images and with a minimum of 1.5 fold change between active and torpid state were statistically analysed using a non-parametric Wilcoxon Rank Sum test (alpha: p < 0.05) with the Delta2D statistic software TMeV (Decodon).

### Preparative 2-D gel for protein identification

Preparative gel separation was employed using pooled samples of all 14 individuals. For the first dimension, unlabeled pooled plasma (total protein concentration = 240 μg) was loaded onto an IPG stripe and separated according to isoelectric points as described above. Separation according to molecular weight in the second dimension was also performed as described above with the exception that a 28 × 21 cm hinged glass cassette (Hoefer Inc., USA) was used instead of low fluorescence glass cassettes. After 2-D gel electrophoresis the gel was Coomassie blue dye stained [0.02% Coomassie blue G-250; 5% w/v aluminum sulfate; 10% v/v ethanol; 2% v/v ortho-phosphoric acid in dH_2_O] for 4 h and then destained [10% ethanol; 2% v/v ortho-phosphoric acid in dH_2_O] to remove unbound Coomassie blue^32^. Spots with a fold change difference of ≥1.5 and a statistical significance of p < 0.05 between the active and torpid state were picked for protein identification. It was not possible to pick all differentially expressed spots as not all spots were distinguishable on the Coomassie blue stained gel. Additional spots for a basic survey of the bat plasma proteome were chosen based on a distinct appearance in the preparative gel and co-localization with protein spots that were differentially expressed. Protein spots of interest were picked manually and stored in Eppendorf tubes containing 5% v/v acetic acid (in dH_2_O) at 4 °C until analysed by mass spectrometry.

### Protein identification by mass spectrometry (MS)

Excised gel spots were washed with water, 25 mM ammonium bicarbonate in acetonitrile/water (1:1) and 50 mM ammonium bicarbonate, shrunk by dehydration in acetonitrile and dried in a speed-vacuum centrifuge. The dry gel pieces were re-hydrated in 20 μL of 50 mM ammonium bicarbonate containing 50 ng trypsin (sequencing grade modified, Promega). After incubation at 37 °C overnight, the enzymatic reaction was terminated by addition of 20 μL of 0.5% (v/v) trifluoroacetic acid in acetonitrile, the liquid was separated, evaporated to dryness under vacuum, and the tryptic peptides were re-dissolved in 6 μL 0.1% (v/v) trifluoroacetic acid , 5% (v/v) acetonitrile in water.

MALDI mass spectrometry was performed as previously described^33^. In brief, the peptides were purified on a C18 RP minicolumn (ZipTip C18, Millipore, Bedford, MA) and eluted directly onto the MALDI target plate using alpha-cyano-hydroxycinnamic acid matrix solution. MS and MS/MS measurements were performed using a MALDI-TOF-TOF instrument (AB SCIEX TOF/TOF 5800; Applied Biosystems, Framingham, MA, USA) equipped with a Neodymium-doped yttrium lithium fluoride laser (Nd:YLF, 349 nm). MS spectra were acquired in positive ion reflector mode by accumulating 5000 consecutive laser shots. For MS/MS, a maximum of 20 precursor ions were selected automatically. GPS Explorer (version 3.6, Applied Biosystems) was used to process the spectra.

LC-MS/MS analyses were performed on an LTQ-Orbitrap XL mass spectrometer (Thermo Fisher) equipped with an Ultimate 3000 nanoLC system (Thermo Scientific). For separation of tryptic peptides, a capillary column (PepMap100, C18, 3 μm, 100 Å, 250 mm × 75 μm i.d., Thermo Scientific) was used. Elution was performed at a flow rate of 300 nL/min using a gradient of 3–50% B in 30 min. Mobile phase A contained 0.1% formic acid in water, and mobile phase B contained 0.1% formic acid in acetonitrile. Mass spectra were acquired in a data-dependent mode with one MS survey scan (with a resolution of 60,000) in the Orbitrap and MS/MS scans of the five most intense precursor ions in the linear trap quadrupole. The dynamic exclusion time for precursor ions was set to 90 s and automatic gain control was set to 1 × 10^6^ for Orbitrap-MS and 10,000 for LTQ-MS/MS scans. The Mascot Distiller Quantitation Toolbox (Matrix Science) was used to generate peak lists.

The processed MS data were analysed on a MASCOT (mass spectral search algorithm) server (version 2.2.2, Matrix Science Ltd, London) and searched in-house against the mammalian subset of the NCBI database (version 221013; 33,055,681 sequences). For MALDI-MS, the mass tolerance of precursor and sequence ions was set to 100 ppm and 0.35 Da, respectively. For LC-MS/MS, the mass tolerance of precursor and sequence ions was set to 10 ppm and 0.35 Da, respectively. A maximum of two missed cleavages was allowed. Methionine oxidation and the acrylamide modification of cysteine were used as variable modifications. A protein was accepted as identified if the total MASCOT score was greater than the significance threshold and at least two peptides appeared the first time in the report and were the top ranking peptides (peptide matches of all proteins identified are listed in supplementary Table S2). For MALDI data, the protein score was −10*log(p), where p is the probability that the observed match is a random event, e.g. protein scores greater than 75 are significant(p < 0.05). For LC-MS/MS data, the ions score was −10*log(p), where p is the probability that the observed match is a random event, e.g. individual ions scores >41 indicate identity or extensive homology (p < 0.05).

## Results

### Differential protein expression of plasma proteins in *M. myotis*

**Figure 1:**
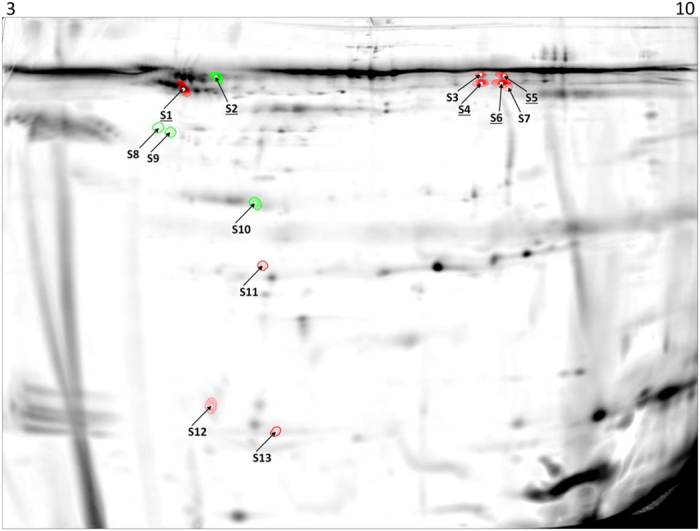
*Myotis myotis* plasma proteome. Fused image of representative images of S-dye labeled *M. myotis* active and torpid plasma samples separated by 2-DIGE. Differential expressed protein spots (p < 0.05; minimum fold-change 1.5) are displayed in either green for up regulated or red for down regulated proteins during torpor and indicated by numbers corresponding to Table 1. Protein spots which were identified via mass spectrometry are underlined. The IPG stripe pH range is indicated on top of the image.

When considering all gel images, we detected a total of 204 matched protein spots present in all individual samples by using a consensus spot pattern. Of 204 protein spots, 13 protein spots (6.4%) showed a significant (p < 0.05) differential expression with a minimum of a 1.5 fold difference between the two physiological states, nine protein spots being down regulated and four being up regulated in torpor compared to the active state (Fig. 1 and Table 1).

We were not able to determine and pick all differential expressed protein spots on the Coomassie stained gel. Protein identification (IDs) were successful for 5 out of 13 differential expressed protein spots (S1/C7, S4/C15, S5/C16, S6/C17 down regulated; S2/C3 up regulated), in which MS data obtained multiple protein IDs for 3 protein spots (S1/C7, S5/C16, S6/C17) and single protein IDs for 2 protein spots (S2/C3, S4/C15). For multiple protein IDs, the top 3 ranked MS peptide matches are shown in Table 1, excluding matches to serum albumin.

Protein matches to those identified in *Myotis davidii* and *Myotis brandtii* based on a MASCOT search of the NCBI database. The significantly down regulated proteins during the torpid state were identified as vitamin D-binding protein, vascular non-inflammatory molecule 3, anti-thrombin III (multiple protein IDs of spot S1/C7), serotransferrin (S4/C15; S5/C16; S6/C17), ß-chain of fibrinogen (S6/C17), and alpha-fetoprotein (S5/C16). The significantly up regulated protein in torpor (S2/C3) was identified as Kininogen-1 (Table 1).

### Survey of expressed plasma proteins in *M. myotis*

**Figure 2:**
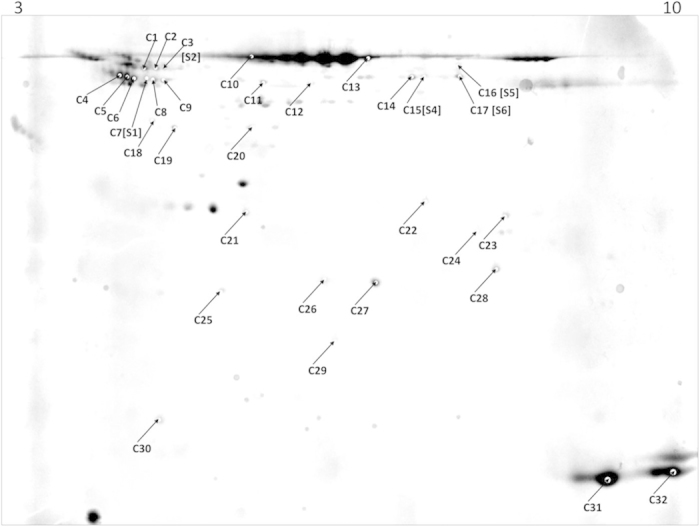
Coomassie Gel of *Myotis myotis* plasma proteome. Preparative Coomassie gel of all 14 plasma samples (pooled) separated by two-dimensional gel electrophoresis. The IPG stripe pH range is indicated on top of the image. Protein numbers display spots that were picked based on co-localization with hibernation specific differentially expressed protein spots distinct appearance in the preparative gel.

To describe a general proteomic profile of bat plasma, 27 protein spots co-localizing with differentially expressed protein spots were picked for analysis (Fig. 2). For 18 out of 27 picked protein spots, MS data yielded multiple protein IDs. The top 3 ranks of MS peptide match results are shown in Table 2 excluding matches to serum albumin. Identified proteins matched those identified in *Myotis davidii* and *Myotis brandtii* based on MASCOT searches of the NCBI protein database. In total, MS yielded 20 different protein IDs of which 12 protein IDs appeared in more than one protein spot. Protein IDs appearing in numerous protein spots included alpha-fetoprotein (20 protein spots (PS)), serotransferrin (7 PS), anti-thrombin III (6 PS), vitamin D-binding protein (6 PS), hemoglobin subunit beta (5 PS), complement C4A (3 PS), fibrinogen beta chain (3 PS), vascular non-inflammatory molecule 3 (3 PS), alpha-1-antitrypsin (2 PS), hemoglobin subunit alpha (2 PS), hemoglobin alpha chain (2 PS) and kininogen-1 (2 PS). Protein IDs found in single protein spots were kininogen-2 (C1), complement C3 (C13), NSFL1 cofactor p47 (C18), apolipoprotein A-V (C19), chain A profilin-Beta-Actin (C20), carbonic anhydrase 2 (C24), adenine phosphoribosyltransferase (C26), apolipoprotein M (C29) and dihydroorotate dehydrogenase (C30) (Table 2). Protein IDs appearing in more than one protein spot were located in similar gel regions (C2, C4, C6, C8 and C9; C11 and C12) expect protein IDs alpha-fetoprotein, fibrinogen, serotransferrin and hemoglobin subunit beta which were located in protein spots distributed over all gel regions.

## Discussion

We observed differential expression for 13 of 204 detectable spots between the active and torpid state representing 6.4% of the total detected protein spots, a similar proportion observed by targeted serum proteomic analysis in American black bears^21^. The protein gel spot pattern exhibited electrophoretic accumulation of protein spots, suggesting an occurrence of multiple protein isoforms. The plasma protein signature clearly differentiated the torpid state from the active homeothermy which is consistent with observed variation in tissues of other hibernating mammalian species^10^,^11^,^12^,^13^,^14^. Mass spectrometric analysis identified seven differentially regulated proteins. Most of them were down regulated (Vitamin D-binding protein (DBP), Serotransferrin (TF), Vascular non inflammatory molecule 3 (VANIN-3), Alpha-fetoprotein (AFP), Fibrinogen beta chain (FIG-β) and Anti-thrombin III (AT)), whereas Kininogen-1 (KNG1) was up regulated.

Down regulated proteins TF, AFP and DBP are classified as transport proteins based on their primary function of binding essential body metabolites, vitamins or metal ions. The iron binding glycoprotein TF plays an important role in cellular and systemic iron homeostasis particularly during long fasting periods^34^. DBP and AFP belong to the albumin gene family in humans and have multifunctional roles in plasma. DBP is a major transporter of Vitamin D3 and its metabolites, and is found as a free plasma protein and also on the surface of many cell types including blood cells^35^,^36^. In contrast, AFP occurs primarily as a free plasma protein in numerous polymeric forms^37^. AFP is involved in the binding and transport of several metabolites including fatty acids, bilirubin and estrogens or metal ions in human fetuses and other mammals^37^,^38^,^39^. The down regulation of these transport proteins might reflect the decreased metabolic rate of hibernating species^3^. Consistent with this hypothesis, studies on diet-restricted rats demonstrated lower mRNA levels of DBP and a decrease of expressed DBP in the liver during fasting^40^,^41^. Furthermore TF and DBP are involved in the modulation of innate immunity^42^,^43^,^44^ and DBP has a particularly important role in the activation of macrophages^45^. Therefore, down regulation suggests a reduction of innate immunity during hibernation^4^.

VANIN-3 is an amidohydrolase involved in the catabolism of CoenzymeA (CoA) from pantothenic acid (Vitamin B5). CoA, and its thioester form acetyl-CoA, are essential cofactors in maintaining fatty acid balance^46^. During hibernation, a switch from glycolysis to the oxidation of triacylglycerols is observed^14^ making fatty acids of adipose tissue the primary energy source. However, the function of VANIN-3 in CoA catabolism is poorly understood. Down regulation during hibernation might suggest an alternate catabolism of CoA reflecting the switch from glucose to triacylglycerols as an energy source.

Differentially expressed proteins FIG-β, AT and KNG1 are part of the blood coagulation system. FIG-β together with other protein domains forms the soluble glycoprotein fibrinogen which is converted by thrombin into insoluble fibrin during formation of blood clots^47^. The serine protease inhibitor AT degrades proteases of the coagulation cascade in order to regulate coagulation and to prevent thrombosis^48^. KNG1 in contrast is part of the kallikrein-kinin system and is known to be essential in many pathways including thrombosis, vascular permeability, and inflammation^49^. During hibernation, platelet aggregation is reduced in brown bears (*Ursus arctos*)^50^. Also an elevation of the protease inhibitor alpha-2-macroglobulin in serum of hibernating ground squirrels and black bears was observed, consistent with reduced coagulation^21^,^51^. In addition, KNG1 was found to be down regulated in hibernating black bears^21^. A decrease of coagulation activity during hibernation could protect the individual from blood clotting during periods of low heart rate and reduced blood flow^1^,^52^. However, the expression pattern of down regulated AT and up regulated KNG1 in *M. myotis* could suggest an elevated coagulation capacity. Similarly, up regulation of coagulation associated genes have been described in hibernating *Myotis brandtii* at the transcription level^25^. In contrast a down regulation of FIG-β during torpor in *M. myotis* may result in a reduction of coagulation capacity^53^. Based on the contradictory findings of proteins involved in coagulation in *M. myotis* we conclude that the detected coagulation associated proteins might be involved in other physiological processes relevant to hibernation. This may imply that bats have a different coagulation cascade compared to other mammals as it is unlikely that increased coagulation during hibernation would be advantageous.

In order to establish a basic plasma proteomic profile in bats, twenty-seven clearly definable protein spots co-localizing with hibernation specific differentially expressed protein spots were identified. MS protein identification yielded multiple IDs per protein spot (Table 2) and IDs found in more than one protein spot including differential expressed protein spots suggesting the co-occurrence of different isoforms, which can be regulated differentially^54^. Most proteins identified are known to be the most abundant proteins in human plasma^16^ including TF, FIG-β, alpha-1-antitrypsin and complement C3. Based on their primary functions, further identified proteins could be categorized as transport proteins (e.g. hemoglobin, apolipoprotein A-V and M), coagulation proteins (e.g. AT, kininogen I and II), proteins of the complement system (e.g. complement factor C4A and C3) and proteins involved in a variety of regulative processes (e.g. carbonic anhydrase). All identified proteins in *M. myotis* can be found in a general plasma profile of humans^16^ indicating similarities in the plasma protein composition across mammalian taxa. However, AFP exhibited an unusual profile by identification in 21 of 34 picked protein spots suggesting a high abundance in plasma of adult *M. myotis*. In humans, AFP is the most abundant protein during fetal development and has a similar function to serum albumin in adults including the binding of hydrophobic ligands such as fatty acids, metabolites and as well metal ions^35^,^36^,^37^. The abundance of AFP in the adult bat plasma suggests that this protein may play an important functional role in different mechanisms in *M. myotis*, maybe in bats in general, thus deserving further scientific attention.

Protein identification was hindered by the lack of protein databases for non-model, wildlife species. Homologous proteins of other species do not always share 100% sequence similarity with unknown *M. myotis* proteins, reducing the amount of potential analysable tryptic peptides per protein depending on the level of similarity which influences the peptide match ranking. This lack of knowledge will certainly change as proteomic research on non-model species develops further^55^. Nonetheless, we were able to establish a basic plasma proteome profile for *M. myotis*. Moreover, we showed differential expression of plasma proteins in hibernating bats compared to active bats demonstrating a modulation of proteins involved in transport, fuel switching from carbohydrate to triacylglycerol oxidation, innate immunity and blood coagulation cascade. In addition protein identification of further protein spots demonstrated evidence for an alternate composition of high abundant plasma proteins in *M. myotis* with AFP as a possible prominent protein in adult bat plasma. Chiropteran hibernation proteome profile was generally consistent with other hibernating species at the pathway level as hypothesized except for coagulation which appears to be myotid bat specific. Further comparisons with rodents and bears will clarify the general similarities and differences among hibernating species proteomic profiles particularly as protein databases improve and identification becomes facilitated.

## Additional Information

**How to cite this article**: Hecht, A. M. *et al.* Plasma proteomic analysis of active and torpid greater mouse-eared bats (*Myotis myotis*). *Sci. Rep.* **5**, 16604; doi: 10.1038/srep16604 (2015).

## Supplementary Material

Supplementary Figure S1

Supplementary Table S2

## Figures and Tables

**Table 1:** Differential protein expression in the *Myotis myotis* plasma proteome.

Spotnumber	FoldChange	*p*value	Protein ID	MSproteinscore	MSmethod	Accessionnumberǂ
S1 [C7]	−1.7	0.012	Vitamin D-binding protein	615	2	gi432093474
			Vascular non inflammatory molecule	566		gi521021447
			Anti-thrombin III	564		gi432097679
S2 [C3]	+2.4	0.001	Kininogen-1	103	1	gi521019686
S3*	−2.2	0.001	*no protein picking possible*	/	/	
S4 [C15]	−2.9	0.001	Serotransferrin	490	1	gi432108417
S5 [C16]	−2.4	0.002	Serotransferrin	457	1	gi432108417
			Alpha-fetoprotein	242		gi521031276
S6 [C17]	−3.3	0.001	Serotransferrin	215	1	gi432108417
			Fibrinogen beta chain	84		gi521022331
S7*	−1.6	0.044	*no protein picking possible*	/		/
S8*	+5.9	0.001	*no protein picking possible*	/		/
S9*	+3.1	0.005	*no protein picking possible*	/		/
S10*	+2.6	0.045	*no protein picking possible*	/		/
S11*	−8.0	0.033	*no protein picking possible*	/		/
S12*	−1.5	0.024	*no protein picking possible*	/		/
S13*	−6.6	0.015	*no protein picking possible*	/		/

Protein spots exhibiting differential expression (p < 0.05; minimum fold-change 1.5) using Delta2D software are shown. Fold change reflects differences in protein spot volume comparing active to torpid samples. Identified proteins via MS techniques MALDI-TOF/TOF (1) and LC-MS/MS (2) are displayed with the total MS protein score based on MASCOT searches on NCBI database and affiliated accession numbers of *Myotis davidii* and *Myotis brandtii* (ǂ). Protein IDs listed are the top 3 ranked protein matches based on the MASCOT score excluding protein matches of serum albumin. Spots S3, S7, S8, S9, S10, S11, S12 and S13 were not possible to relate to a protein spot on preparative gels and thus could not be picked and identified (*).

**Table 2:** Identified proteins in *Myotis myotis* plasma proteome.

Spotnumber	Protein ID	MSproteinscore	MSmethod	Accessionnumberǂ
C1	Kininogen-1	84	1	gi432105324
	Kininogen-2	60		gi521032202
C2	Vitamin D-binding protein	690	2	gi432093474
	Antithrombin-III	594		gi432097679
	Alpha-fetoprotein	550		gi521031276
C3 [S2]	Kininogen-1	103	1	gi432105324
C4	Alpha-1-antitrypsin	633	2	gi432096197
	Antithrombin-III	461		gi432097679
	Vitamin D-binding protein	434		gi432093474
C5	Alpha-1-antitrypsin	374	1	gi432096197
C6	Vitamin D-binding protein	658	2	gi432093474
	Antithrombin-III	607		gi432097679
	Alpha-fetoprotein	545		gi521031276
C7 [S1]	Vitamin D-binding protein	615	2	gi432093474
	Vascular non-inflammatory molecule 3	566		gi521021447
	Antithrombin-III	564		gi432097679
C8	Vitamin D-binding protein	494	1	gi432093474
	Vascular non-inflammatory molecule 3	55		gi521021447
C9	Vitamin D-binding protein	425	1	gi432093474
	Vascular non-inflammatory molecule 3	76		gi521021447
C10	Alpha-fetoprotein	299	1	gi521031276
C11	Alpha-fetoprotein	724	2	gi521031276
	Anti-thrombin III	576		gi432097679
	Fibrinogen beta chain	566		gi521022331
C12	Alpha-fetoprotein	587	2	gi521031276
	Anti-thrombin III	506		gi432097679
	Serotransferrin	394		gi432108417
C13	Alpha-fetoprotein	1111	2	gi521031276
	Serotransferrin	819		gi432108417
	Complement C3	535		gi521031112
C14	Fibrinogen beta chain	677	1	gi521022331
C15 [S4]	Serotransferrin	490	1	gi432108417
C16 [S5]	Serotransferrin	457	1	gi432108417
	Alpha-fetoprotein	242		gi521031276
C17 [S6]	Serotransferrin	215	1	gi432108417
	Fibrinogen beta chain	84		gi521022331
C18	Alpha-fetoprotein	596	2	gi521031276
	Complement C4-A	462		gi432089459
	NSFL1 cofactor p47	339		gi431894242
C19	Alpha-fetoprotein	614	2	gi521031276
	Complement C4-A	356		gi432089459
	Apolipoprotein A-V	329		gi432105735
C20	Alpha-fetoprotein	969	2	gi521031276
	Complement C4-A	534		gi432089459
	Chain A, Profilin-Bet-Actin	487		gi313507212
C21	Alpha-fetoprotein	175	1	gi521031276
C22	Alpha-fetoprotein	987	2	gi521031276
	Serotransferrin	786		gi432108417
	Hemoglobin, subunit beta	296		gi432107589
C23	Alpha-fetoprotein	166	1	gi521031276
C24	Carbonic anhydrase 2	88	1	gi432088987
	Alpha-fetoprotein	87		gi521031276
C25	Alpha-fetoprotein	303	1	gi521031276
C26	Alpha-fetoprotein	720	2	gi521031276
	Hemoglobin, subunit beta	356		gi432107589
	Adenine phosphoribosyltransferase	314		gi432104870
C27	Alpha-fetoprotein	739	2	gi521031276
	Hemoglobin, subunit beta	308		gi432107589
	Serotransferrin	268		gi432108417
C28	Alpha-fetoprotein	109	1	gi521031276
C29	Alpha-fetoprotein	297	2	gi521031276
	Hemoglobin, subunit beta	277		gi432107589
	Apolipoprotein M	217		gi432089435
C30	Hemoglobin, subunit beta	355	2	gi432107589
	Alpha-fetoprotein	317		gi521031276
	Dihydroorotate dehydrogenase	257		gi521035253
C31	Hemoglobin subunit alpha	347	1	gi110831911
	Hemoglobin alpha chain	274		gi189909345
C32	Hemoglobin subunit alpha	356	1	gi122428
	Hemoglobin alpha chain	335		gi189909345

Protein IDs obtained using MS techniques MALDI-TOF/TOF (1) and LC-MS/MS (2) are displayed with the total MS protein score based on MASCOT searches on the NCBI database and affiliated accession numbers of *Myotis davidii* and *Myotis brandtii* (ǂ). Protein IDs listed are the top 3 ranked protein matches based on the MASCOT score excluding protein matches to serum albumin.
