# Supplementary figures and images for: Plasma proteomic analysis of active and torpid greater mouse-eared bats (Myotis myotis)
Source: Sci Rep. 2015 Nov 20;5:16604. doi: 10.1038/srep16604 (PMC4653738; doi:10.1038/srep16604)

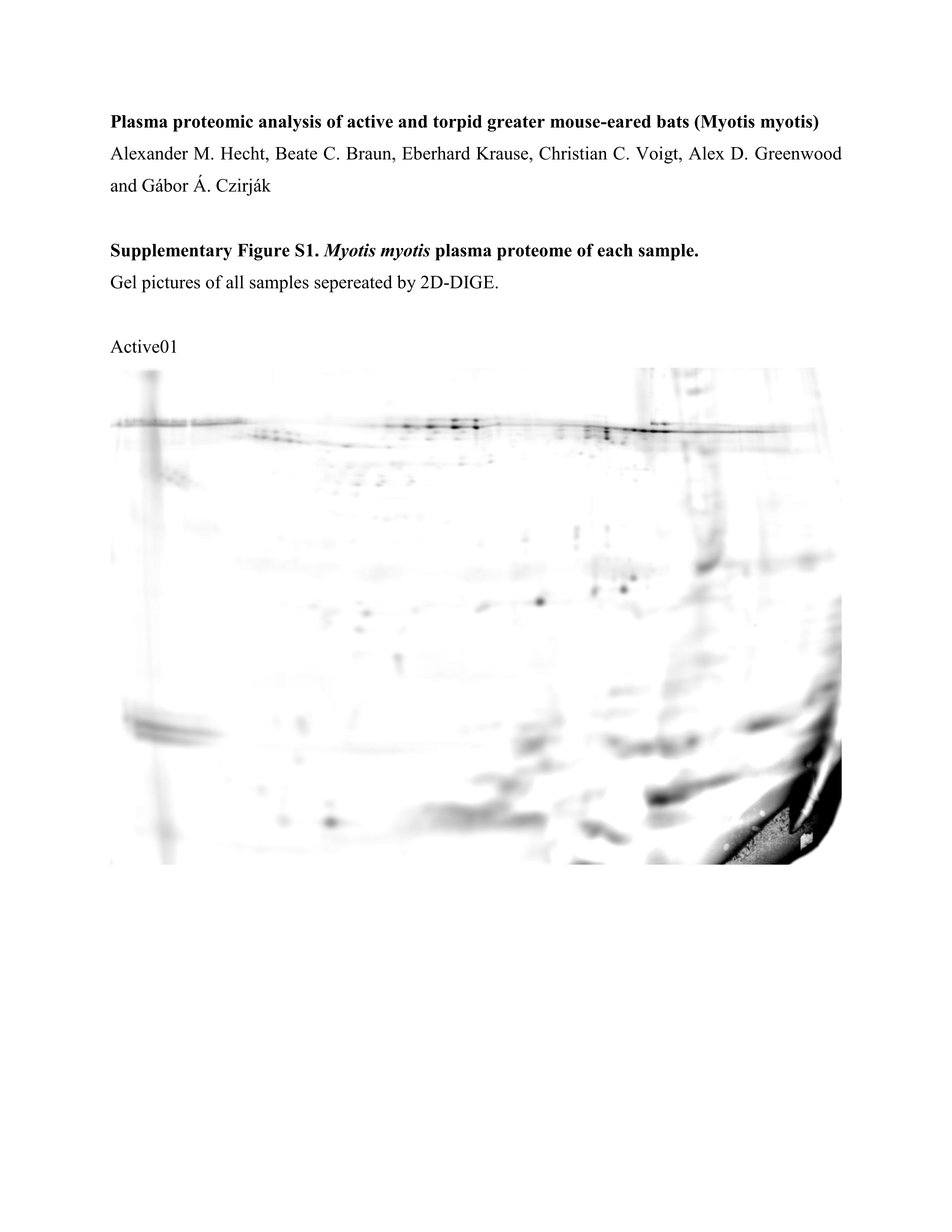

Supplement: Supplementary Figure S1 [file srep16604-s1.jpg]
